# NF-кB c-Rel modulates pre-fibrotic changes in human fibroblasts

**DOI:** 10.1007/s00403-021-02310-2

**Published:** 2021-12-09

**Authors:** Lara Carolina Micus, Franziska Susanne Trautschold-Krause, Anna Lena Jelit, Michael Peter Schön, Verena Natalie Lorenz

**Affiliations:** Department of Dermatology, Venereology and Allergology, University Medical Center Göttingen, Göttingen Lower Saxony, Robert Koch Str. 40, 37075 Göttingen, Germany

**Keywords:** NF-κB c-Rel, Skin fibrosis, Contraction, N-cadherin, Dermal fibroblasts

## Abstract

Skin fibrosis is one central hallmark of the heterogeneous autoimmune disease systemic sclerosis. So far, there are hardly any standardized and effective treatment options. Pathogenic mechanisms underlying fibrosis comprise excessive and uncontrolled myofibroblast differentiation, increased extracellular matrix protein (ECM) synthesis and an intensification of the forces exerted by the cytoskeleton. A deeper understanding of fibroblast transformation could help to prevent or reverse fibrosis by specifically interfering with abnormally regulated signaling pathways. The transcription factor NF-κB has been implicated in the progression of fibrotic processes. However, the cellular processes regulated by NF-κB in fibrosis as well as the NF-κB isoforms preferentially involved are still completely unknown. In an in vitro model of fibrosis, we consistently observed the induction of the c-Rel subunit of NF-κB. Functional abrogation of c-Rel by siRNA resulted in diminished cell contractility of dermal fibroblasts in relaxed, but not in stressed 3D collagen matrices. Furthermore, directed migration was reduced after c-Rel silencing and total N-cadherin expression level was diminished, possibly mediating the observed cellular defects. Therefore, NF-кB c-Rel impacts central cellular adhesion markers and processes which negatively regulate fibrotic progression in SSc pathophysiology.

**Supplementary Information:**

The online version contains supplementary material available at 10.1007/s00403-021-02310-2.

## Introduction

Fibrosis leads to fundamental pathological tissue remodeling, a central element of which is the accumulation of several proteins of the extracellular matrix (ECM) [1]. While fibrosis can occur in a variety of diseases, systemic sclerosis is a prototypical fibrotic disease that is associated with dysregulated immune functions and vascular changes [2, 3]. Long-standing fibrosis can also give rise to malignant transformation [4].

An important trigger of fibrosis is the transforming growth factor (TGF)-β1. It mediates activation of key pathways such as Smad signaling and induces fibrotic markers as well as extracellular matrix protein secretion [5]. The mechanisms of skin fibrosis involve a marked proliferation of dermal fibroblasts and their differentiation into contractile myofibroblasts [6]. Myofibroblasts mediate fibrosis-associated tissue reformation through cytoskeletal rearrangements and alpha smooth muscle actin (αSMA) integration. Furthermore, myofibroblasts upregulate the production of ECM proteins such as collagen type I and III, the F-actin-binding protein SM22α and the serine protease inhibitor plasminogen activator-inhibitor (PAI)-1 [7, 8]. Physiologically, myofibroblasts contract wounds and they dissolve by apoptosis. However, for reasons still unknown, this apoptosis does not work in fibrosis [9, 10]. Current antifibrotic therapies aim to target myofibroblasts by small molecule inhibitors affecting Smad or Wnt/β-catenin signaling [11].

The transcription factor NF-κB is a major player in immunity, tumorigenesis and other processes. It mediates signals that are for example of fundamental importance for proliferation and apoptosis [12, 13]. It is activated as homo- or heterodimer of five proteins, p50, p65, c-Rel, p52 and RelB. In fibrosis, NF-κB (p65) activity is increased in dermal and lung fibroblasts [14]. The p65 subunit inhibits collagen I expression in dermal fibroblasts [15], and p50 appears to be a genetic risk locus for systemic sclerosis (SSc) [16, 17].

In connection with fibrosis, the c-Rel subunit has become the focus of scientific interest: *c-rel*^*−/−*^ mice were protected against bleomycin-induced fibrosis of the skin [18]. Similarly, c-Rel seems to protect against fibrosis in the liver [19] and the heart [20].

Furthermore, *c-rel*^*−/−*^ mice showed B- and T-cell defects [21, 22], particularly in regulatory T-cells [23]. Finally, c-Rel also participates in the regulation of the cell cycle and mitosis in keratinocytes and some epithelial tumor cells [24–26].

Since no satisfactory treatment for fibrosis is yet available, a deeper understanding of underlying molecular processes could contribute to the development of such a treatment.

In our study, fibrotic stimulation of human dermal fibroblasts specifically induced c-Rel. Silencing of c-Rel by siRNA led to decreased contractility of non-stressed collagen matrices, whereas contractility of stressed matrices remained unaffected. While viability of dermal fibroblasts remained unchanged, their directed migration was significantly reduced when c-Rel was suppressed. Regarding adhesion marker expression, prominent suppression of N-cadherin resulted, typically upregulated in fibrosis and many cancer types. Thus, c-Rel takes part in the homeostasis of dermal fibroblasts and seems to regulate some steps of fibrotic activation.

## Materials and methods

### Antibodies

Primary antibodies directed against the following antigens were used: p50 (Abcam, Cambridge, USA), p65 (Cell Signaling Technology, Danvers, USA), c-Rel (Cell Signaling Technology), RelB (Santa Cruz Biotechnology, Dallas, USA) and p52 (Cell Signaling Technology), αSMA (Abcam), PAI-1 (Santa Cruz Biotechnology), pSmad3 (Abcam), GAPDH (Cell Signaling Technology), focal adhesion kinase, FAK (Abcam), SM22α (ThermoFisher Scientific, Waltham, USA), talin (Abcam), vinculin (SigmaAldrich, St. Louis, USA), actin (Merck Millipore, Burlington, USA), N-cadherin (Abcam) and Calnexin (ENZO Life Sciences, Belgium).

### Cell culture and stimulation

BJ dermal fibroblasts (ATCC^®^ CRL-2522™) were cultured in EMEM (SigmaAldrich) growth media supplemented with 10% FCS (Biochrom, Berlin, Germany) and 1% l-glutamine (Lonza, Morristown, USA) at 37 °C with 5% CO_2_ and were frequently tested for mycoplasma contamination. Cells were stimulated after adherence with rh-TGF-β1 (10 ng/ml, ThermoFisher Scientific).

### siRNA transfection

Adherent fibroblasts were transfected using Promofectin siRNA (Promocell, Heidelberg, Germany) with c-Rel siRNA I (SI00045570), c-Rel siRNA II (SI03070599) and AllStars Negative Control siRNA (Qiagen, Hilden, Germany) at 40 nM for 72 h. Untreated and transfection reagent-treated controls were performed to exclude nonspecific effects.

### Collagen gel contraction assay

Collagen gels populated with 4 × 10^4^ transfected fibroblasts per well were prepared using a final concentration of 1 mg/ml collagen I (ThermoFisher Scientific) in growth medium and polymerized using 1 M NaOH (Merck, Billerica, USA). Polymerized gels where covered with growth medium and either detached directly (relaxed) or adhered for 24 h including TGF-β stimulation (stressed) followed by final photographic documentation using a LAS4000 device (Fujifilm, Tokyo, Japan). Quantification of collagen gel size was determined using ImageJ Vers. 1.52 (NIH, USA).

### Cell migration assay

On each side of the migration insert, 3.5 × 10^4^ transfected cells were seeded on collagen I-coated culture inserts (Ibidi, Gräfelfing, Germany) in medium with low serum concentration (2.5% FCS). Migration into the cell-free gap was documented for indicated time points using an Axiovert200 microscope and the Axiovision software (Zeiss, Jena, Germany). Quantification of the cell-free gap was performed using ImageJ Vers. 1.52 (NIH, USA).

### G/F actin fractionation

The G/F actin ratio was determined using the G actin/F actin kit as recommended by the manufacturer (Cytoskeleton Inc., Denver, USA) and analyzed by western blotting. Quantification was performed using ImageJ vers. 1.52 (NIH, USA).

### Immunofluorescence

In each well, 6 × 10^3^ transfected cells were cultured in 8 well chamber slides (ThermoFisher Scientific) followed by fixation with 4% paraformaldehyde. After blocking/permeabilization with 5% FCS/0.25% Triton-X/PBS, primary antibodies were incubated followed by anti-mouse Alexa488 (Cell Signaling Technology) and anti-rabbit Alexa 555 (ThermoFisher Scientific) labeled secondary antibodies. Finally, cells were embedded in Fluorescent Mounting Medium (Dako, Santa Clara, USA) supplemented with 1 µg/ml DAPI (SigmaAldrich). Images were acquired using AxioImager M1 and the Axiovision Software Rel. 4.7.1 (Zeiss).

### RNA isolation, cDNA synthesis and qRT-PCR

RNA was isolated using InnuPrep RNA kit (Analytik Jena, Jena, Germany) and cDNA was generated using SuperScript IV cDNA Synthesis Kit (ThermoFisher Scientific). For qPCR, Eva Green Dye (Solis Biodyne, Tartu, Estonia) was used and respective primer pairs; p50 (fw: 5′-CACTTAGCAATCATCCACCTT-3′, rev: 5′-AGCCCTCAGCAAATCCT-3′), p65 (Qiagen; QT02324308), c-Rel (Qiagen; QT00052472), p52 (fw: 5′-GGGGCATCAAACCTGAAGATTTCT-3′, rev: 5′-TCCGGAACACAATGGCATACTGT-3′), RelB (Qiagen QT00038640), CTGF (fw: 5′-CTCGCGGCTTACCGACTG-3′, rev: 5′-GGCTCTGCTTCTCTAGCCTG-3′), PAI-1 (SERPINE-1) (fw: 5′-CTCTCTCTGCCCTCACCAAC-3′, rev: 5′-GTGGAGAGGCTCTTGGTCTG-3′) αSMA (fw: 5′-CGTGGGTGACGAAGCACAG-3′, rev: 5′-GGTGGGATGCTCTTCAGGG-3′), collagen IAI (fw: 5′-GCTCCTGCTCCTCTTAGCG-3′, rev 5′-CCGTTCTGTACGCAGGTGAT-3′), RNA-polymerase IIA (fw: 5′-GGAGATTGAGTCCAAGTTCA-3′, rev: 5′-GCAGACACACCAGCATAGT-3′), integrin αv (fw: 5′-CACTTCGGCGATGGCTTTTC-3′, rev: 5′-GTAGCAGGAGTCCCGAGAGA-3′), integrin α2 (fw: 5′-GTGGCTTTCCTGAGAACCGA-3′, rev: 5′-GATCAAGCCGAGGCTCATGT-3′), integrin β1 (fw: 5′-ACGCCGCGCGGAAAAGATGA-3′, rev: 5′-GCACCACCCACAATTTGGCCC-3′), Data analysis was performed using QuantStudio5 and QuantStudio Design and Analysis Software (ThermoFisher Scientific).

### Protein lysates and Western blotting

Cells were resuspended in RIPA lysis and extraction buffer (ThermoFisher Scientific), incubated for 30 min at 4 °C and centrifuged at 14.000*g*. Western blots were performed as described previously [26].

### MTT assay

For viability testing, 5 × 10^3^ cells were seeded and finally measured using the Non-Radioactive Cell Proliferation Assay (Promega, Mannheim, Germany) and determined 72 h after transfection measuring absorbance at 570 nm and 630 nm using Appliskan (ThermoFisher Scientific).

### Statistical analysis

For MTT, migration and collagen contractility assay, one way analysis of variance (ANOVA) was performed followed by post tukey test using Graph Pad PRISM vers. 7 (GraphPad Software, Inc.). For qRT PCR, relative expression software tool (REST) Software (Qiagen, 2009) was used. Differences were considered statistically significant when *P* ≤ 0.05.

## Results

### Fibrotic stimulation with TGF-β1 induces myofibroblastic traits in vitro

**Fig. 1:**
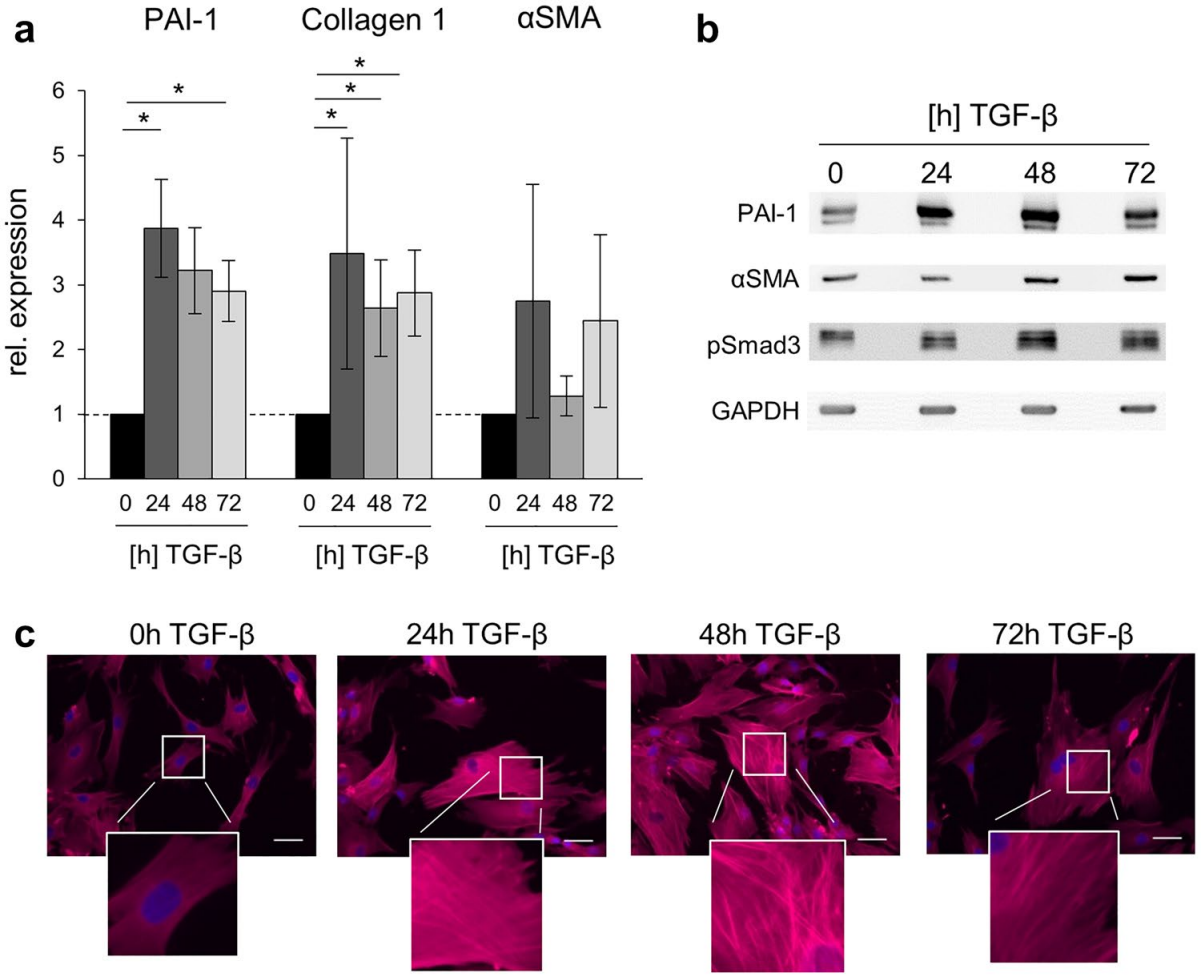
Myofibroblast-related changes are induced by TGF-β stimulation. **a** qPCR of fibrotic markers (PAI-1, Collagen IAI and αSMA) after TGF-β stimulation for 0 h, 24 h, 48 h and 72 h, respectively. Error bars = SEM, * indicates *p* ≤ 0.05, *n* = 3–4 independent experiments. **b** Protein expression of fibrotic markers after 0 h, 24 h, 48 h and 72 h of TGF-β stimulation. One of 3–4 representative western blots is shown, GAPDH served as loading control. **c** Immunofluorescence staining of filamentous actin binding protein SM22α showing morphologic changes after 0 h, 24 h, 48 h and 72 h of TGF-β stimulation, respectively. Scale bar = 50 µm, *n* = 3

In a previously described in vitro model of fibrosis [27, 28], human BJ fibroblasts with extended life span were stimulated with TGF-β1 (10 ng/ml) and their expression of different fibrosis markers was investigated and validated (Fig. 1).

As detected by quantitative RT-PCR, transcription of plasminogen activator inhibitor-1 (PAI-1) was significantly increased (3.87-fold after 24 h, 3.22-fold after 48 h, 2.9-fold after 72 h). Likewise, collagen IAI (3.49-, 2.64- and 2.88-fold, respectively) and alpha-smooth muscle actin (αSMA; 2.75-, 1.28- and 2.44-fold, respectively) were induced, albeit the increase of αSMA did not reach statistical significance (Fig. 1a).

On the protein level, PAI-1 showed the most pronounced and earliest TGF-β associated induction, while αSMA was induced 48 h and 72 h after TGF-β stimulation. Furthermore, pSmad3 expression analysis served as positive control for TGF-β signaling activation and showed clear induction at all time points of stimulation (Fig. 1b).

Finally, we detected fibrosis-associated changes of the cytoskeleton in this model. As demonstrated by staining of the F-actin binding protein SM22α, contractile actin filaments increased markedly following exposure of the cultures to TGF-β (Fig. 1c). Thus, our in vitro fibrosis model reflected key fibrotic and myofibroblast-associated changes.

### NF-κB c-Rel is induced by fibrotic stimulation

**Fig. 2:**
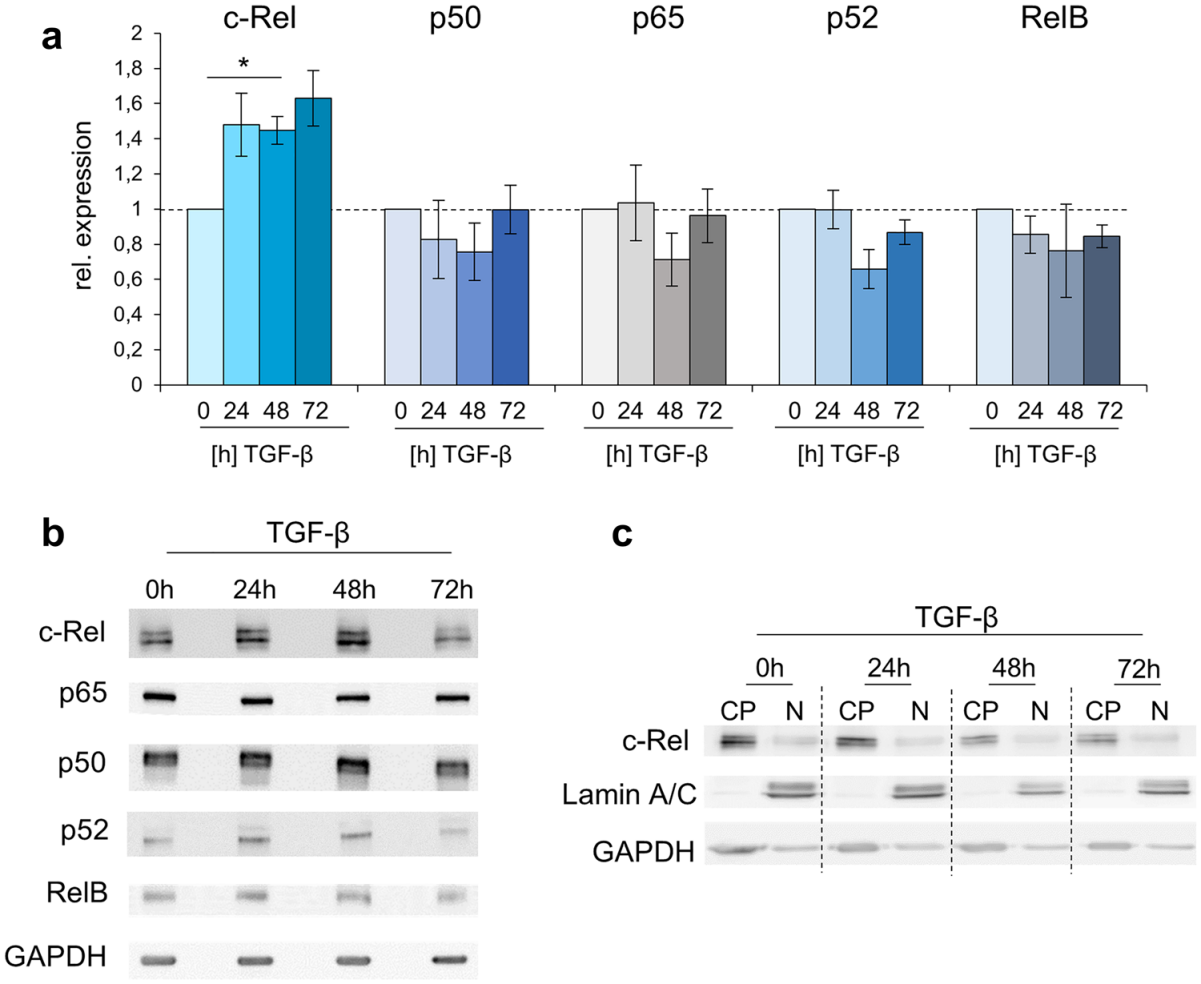
NF-κB expression and activity in fibrotically activated dermal fibroblasts. **a** qPCR of c-Rel, p50, p65, RelB and p52 after TGF-β stimulation for 0 h, 24 h, 48 h and 72 h, respectively. Error bars = SEM, *n* = 3–4, *p ≤ 0.05. **b** Western blotting of c-Rel, p50 and p65, p52 and RelB expression after 0 h, 24 h, 48 h and 72 h TGF-β stimulation. GAPDH served as loading control. One representative experiment of four is shown. **c** Subcellular distribution of c-Rel after TGF-β stimulation for 0 h, 24 h, 48 h and 72 h, respectively. Nuclear (N) and cytoplasmic (CP) lysates are depicted, Lamin A/C and GAPDH served as nuclear and cytoplasmic loading control, respectively. One of four independent experiments is depicted

When expression and activity of NF-κB proteins were analyzed after incubation of BJ fibroblasts with TGF-β1, only c-Rel mRNA consistently showed elevation (by 48%, 45% and 63% after 24 h, 48 h and 72 h, respectively; Fig. 2a). Correspondingly, the c-Rel protein was also more strongly expressed after TGF stimulation with a maximum after 48 h, while p65, p50, p52 and RelB remained unchanged (Fig. 2b). An analysis of subcellular distribution showed that c-Rel was localized both in unstimulated fibroblasts and after TGF-β stimulation mainly in the cytoplasm and considerably less in the nucleus (Fig. 2c).

### c-Rel is involved in the contraction of collagen matrices by dermal fibroblasts

**Fig. 3:**
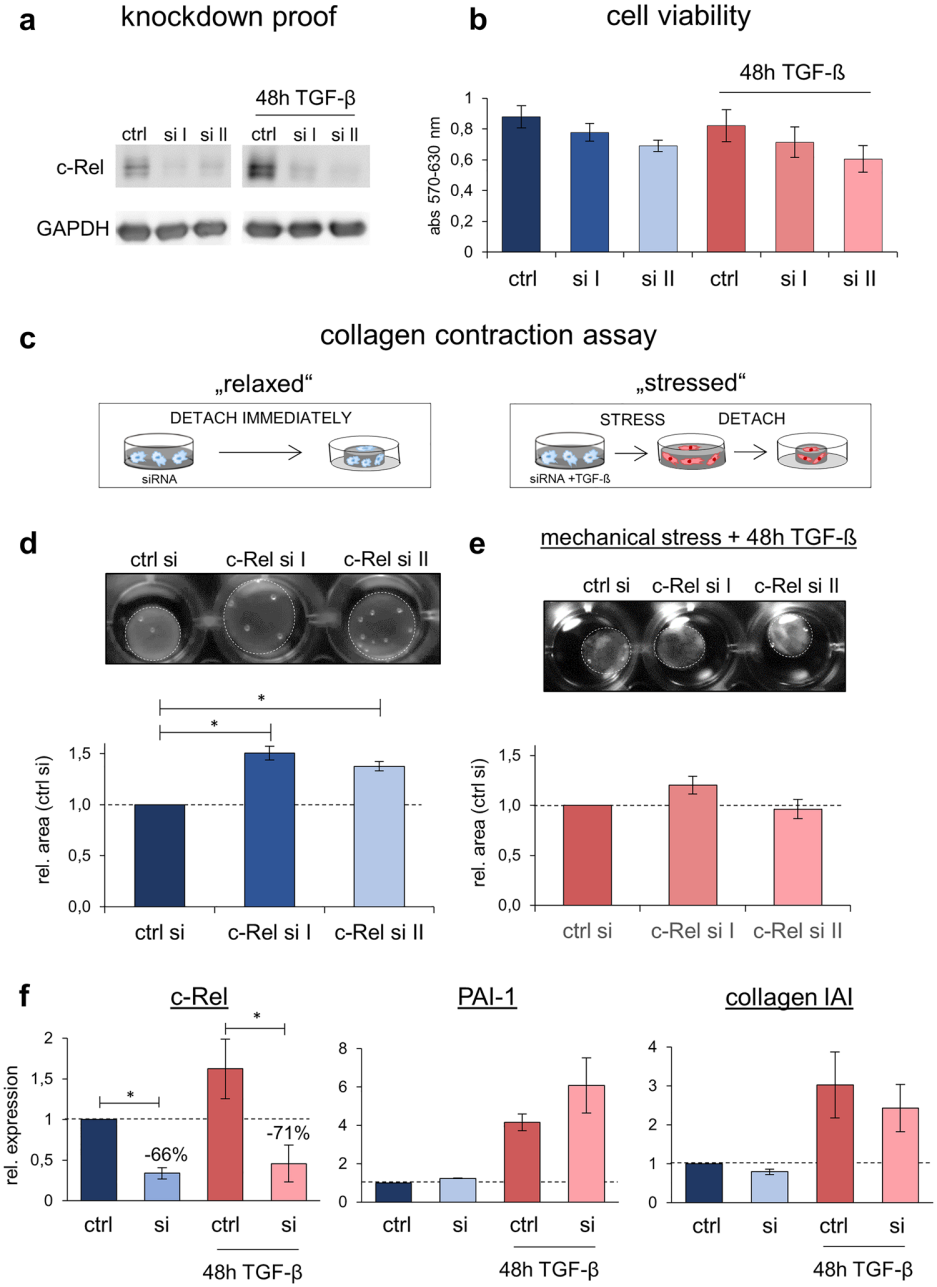
c-Rel siRNA suppression affects cell contractility. **a** Western blotting of ctrl siRNA and c-Rel siRNA (c-Rel I and c-Rel II) transfected samples, either unstimulated (left) or stimulated with TGF-β (right) 72 h after transfection showing efficient c-Rel knockdown. GAPDH served as loading control. **b** MTT viability assay of ctrl, c-Rel I and c-Rel II siRNA transfected cells. The mean absorbance values of three independent experiments are shown (error bars = SEM). **c** Schematic illustration of 3D collagen contraction assay using dermal fibroblasts in a “relaxed” model (left side) and in a “stressed” model by induction of mechanical stress plus TGF-β stimulation (right side). **d**, **e** Collagen contractility assay of ctrl siRNA, c-Rel I and c-Rel II siRNA transfected dermal fibroblasts in “relaxed” (**d**) and “stressed” lattices (**e**) 72 h after siRNA transfection. One representative experiment is depicted while below, quantification of cell-collagen-matrix area is depicted. Relative mean values of 3–4 experiments are shown, error bars = SEM, * indicates *p* ≤ 0.05. **f** qPCR of c-Rel, PAI-1, and collagen IAI of control and c-Rel siRNA transfected dermal fibroblasts 72 h after transfection. Relative mean values of three independent experiments are shown, RNA-polymerase IIA served as reference gene, error bars = SEM, * indicates *p* ≤ 0.05

The data collected so far suggested that c-Rel modulates fibrosis processes. To test this hypothesis further, its activity was suppressed in fibroblasts by transfection with two siRNA constructs directed against c-Rel. Both unstimulated (homeostatic) and fibrotic conditions showed efficient c-Rel downregulation 72 h after treatment and fibroblast viability was not significantly altered after c-Rel suppression with either of the two constructs (Fig. 3a, b). Of note, this downregulation of c-Rel did not lead to compensatory changes of the major dimerization partners, p50 and p65 (supplementary Fig. 1a).

Cytoskeletal remodeling and the exertion of traction forces are essential characteristics of myofibroblastic transdifferentiation. 3D contraction assays using cell-collagen I-matrices were used to mimick these features. Non-stressed conditions were generated by gently releasing the collagen gels (without TGF supplementation) from the vessel wall directly after polymerization (hereinafter referred to as “relaxed” conditions; Fig. 3c left side and Fig. 3d [29]). To simulate fibrotic conditions, the collagen gels were polymerized supplemented with TGF-β and not detached from the vessel wall until 24 h after polymerisation (hereinafter referred to as "stressed" conditions; Fig. 3c right side and Fig. 3e [29]). Following c-Rel suppression, relaxed fibroblast-collagen I-matrices were significantly and reproducibly larger than control matrices (50.6% with c-Rel I siRNA, *p* ≤ 0.05; 37.7% with c-Rel II siRNA, *p* ≤ 0.05; Fig. 3b). In contrast, stressed matrices showed minor differences in contractility after c-Rel suppression (Fig. 3e). Thus, c-Rel seemed to significantly affect fibroblast contractility in relaxed but not in stressed collagen matrices. After siRNA-induced significant suppression of c-Rel, the transcription of fibrotic marker PAI-1 was only moderately induced while collagen 1A1 was slightly reduced and no significant expression changes occurred following TGF-β stimulation (Fig. 3f). Protein expression of pSmad3, PAI-1 and αSMA was not significantly altered (supplementary Fig. 1b).

### Impaired motility in c-Rel suppressed dermal fibroblasts

In addition to contractility, which we had demonstrated to be influenced by c-Rel, altered motility and migration ability of fibroblasts also play an important role in wound healing and fibrosis [30]. In the following experiments, the migration of normal (control transfected) fibroblasts was therefore compared in artificial "wounds" (standardized scratch assays) with that of fibroblasts whose c-Rel was suppressed by siRNA for periods of up to 24 h.

**Fig. 4:**
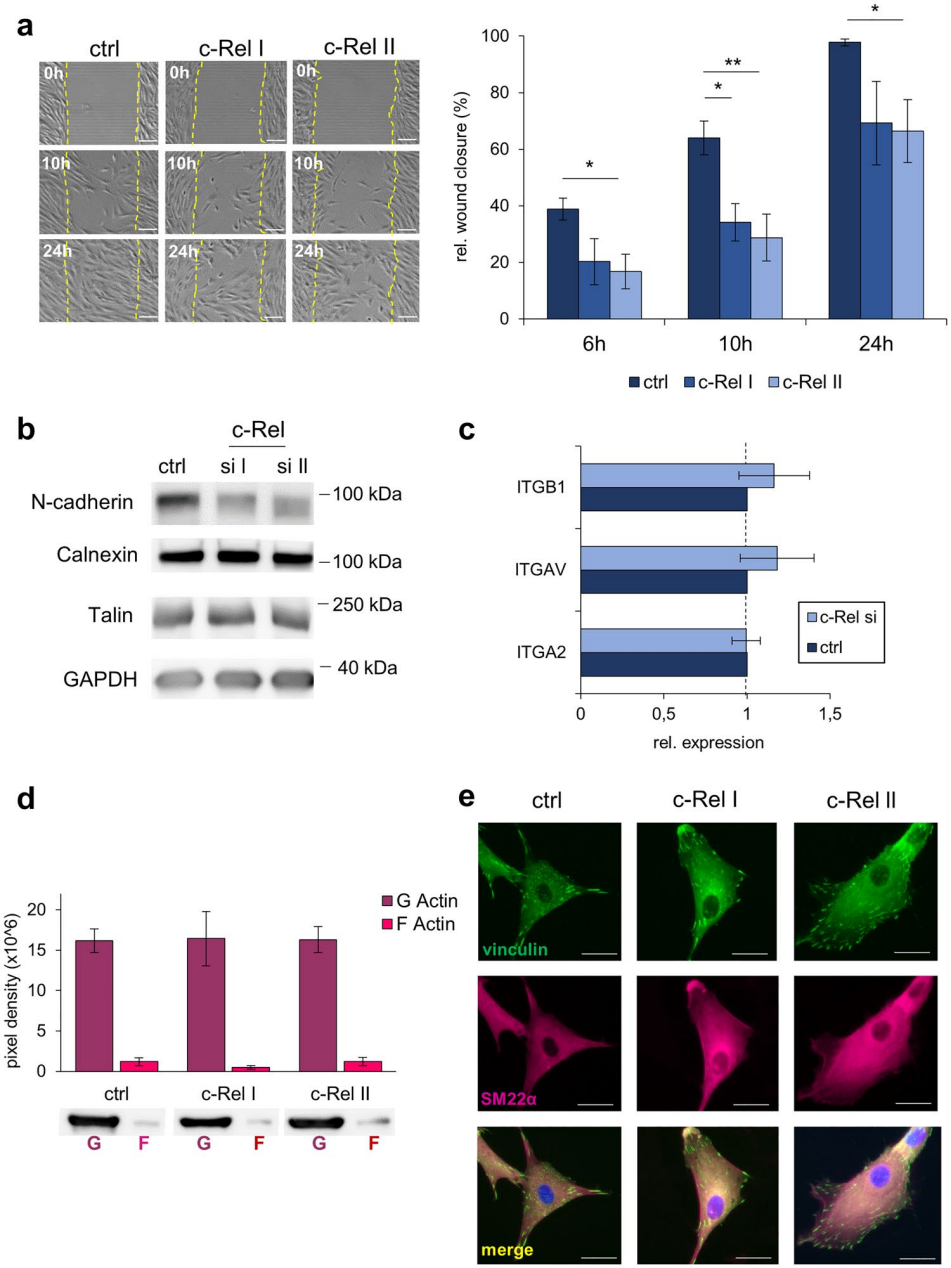
Silencing of c-Rel impairs dermal fibroblast migration and N-cadherin expression. **a** Migration assay of control siRNA (ctrl), c-Rel I siRNA and c-Rel II siRNA transfected fibroblasts. Representative images are shown 0 h, 10 h and 24 h after assay start, quantitative evaluation shows mean values of three independent experiments compared to assay start. Error bars = SEM. *p ≤ 0.05, **p ≤ 0.01. **b** Western blotting of N-cadherin and Talin of control siRNA, c-Rel siRNA I and II 72 h after transfection. One of three experiments is shown, GAPDH and Calnexin served as loading control. **c** qPCR of integrin α_V_ (ITGAV), β_1_ (ITGB1), α_2_ (ITGA2) of control and c-Rel siRNA transfected fibroblasts 72 h after transfection. The mean values of three-four independent experiments are shown (error bars = SEM). **d** G and F actin distribution of control siRNA, c-Rel siRNA I and II transfected cells. The mean pixel intensities of three independent experiments are depicted (error bars = SEM) and one representative western blot experiment is shown below. **e** SM22α and vinculin immunofluorescence of control siRNA, c-Rel I siRNA and c-Rel II siRNA transfected fibroblasts. Single representative cells of one of three independent experiments processed identically are shown. Nuclei were counterstained with DAPI in merged illustrations, size bar = 25 µm

After 6 h, c-Rel suppression reduced directed cell migration by 47.9% for siRNA I and 56.9% for siRNA II compared to control transfected fibroblasts, the latter difference being already statistically significant at this early stage (*p* < 0.05). After 10 h the difference between control cells and the two c-Rel suppressed populations was also consistently detectable (46.6% and 55.0%, respectively; with *p* < 0.05 and *p* < 0.01). The delayed migration of c-Rel-suppressed fibroblasts was still visible after 24 h, with the values of the c-Rel-suppressed populations slowly approaching the already completely closed "wound" of the control cells (*p* < 0.05; Fig. 4a).

To understand the molecular mechanisms, we analyzed expression of adhesion marker N-cadherin and focal adhesion protein talin by western blot analysis and determined strong downmodulation of N-cadherin following c-Rel suppression (Fig. 4b).

However, the expression of the classical adhesion receptor integrin subunits β1 (ITGB1), α2 (ITGA2) and αV (ITGAV) was not significantly altered by c-Rel suppression (Fig. 4c). The significant impairment of N-cadherin expression following c-Rel suppression was also not accompanied by a change in the relative proportions of globular (G) and filamentous (F) actin, as demonstrated by fractional analysis (Fig. 4d). Finally, immunofluorescence staining of vinculin and SM22α showed that also the formation of focal adhesions was not affected by c-Rel suppression (Fig. 4e).

Together, our findings showed that c-Rel suppression interferes with N-cadherin expression potentially disturbing cell contraction and migration, while only marginally, if at all, touching on cytoskeleton or focal adhesion characteristics.

## Discussion

Fibrotic diseases such as systemic sclerosis (SSc) comprise complex pathological tissue alterations, which, in addition to misregulated immune functions and vascular changes, include organ fibrosis in particular [31]. In SSc patients, fibrosis usually starts in the skin and can proceed to organs such as lung, kidney and heart [32]. Therefore, a better understanding of signaling mechanisms in skin-derived fibroblasts may help to develop new therapeutic targets preventing uncontrolled fibrotic proceeding of SSc. While the pathogenesis of fibrosis is still largely enigmatic, the c-Rel subunit of NF-κB appears to be associated with fibrotic changes in various organs such as the skin, liver and heart as suggested by c-Rel-deficient mice [18–20]. However, a direct association of c-Rel with fibrosis-relevant mesenchymal cells has scarcely been investigated so far.

In this situation, we showed induction of c-Rel in fibroblasts in an in vitro fibrosis model both on the transcriptional and the translational level. No other NF-κB subunit was similarly induced in fibroblasts by TGF-β. This is somewhat surprising, since NF-κB as a central transcription factor is involved in numerous regulatory processes [33] and TGF-β can exert rather pleiotropic effects [34]. However, in some respects c-Rel is unusual among NF-кB proteins as it possesses a broader nuclear recognition site and, consecutively, has a larger spectrum of target genes compared to other NF-κB subunits [35]. Moreover, it is thought to be associated with a more pronounced capability to suppress genes in a proinflammatory surrounding [36].

Following suppression of c-Rel, N-cadherin expression was reduced, possibly mediating diminished contractility and migration of human dermal fibroblasts. Supporting our findings, N-cadherin was recently shown to be involved in injury-triggered migration (“swarming”) and contraction of fascia fibroblasts important for scar formation [37].

Subsequent analyses need to reveal whether N-cadherin serves as a direct target of c-Rel and whether also typical intracellular target molecules such as α- and β-catenin are affected. Although N-cadherin and actin cytoskeleton dynamics are intricately intertwined, no changes appeared neither in G/F actin content nor in F-actin signal intensity. Possibly, associated cytoskeletal mechanisms include rather actomyosin-based dynamics [38]. Moreover, *c-rel*^*−/−*^ mice revealed decreased bladder smooth muscle contraction depending on CPI-17 (protein kinase C-potentiated inhibitory protein of 17 kDa), a protein affecting myosin light chain phosphorylation [39]. This suggests an alternative impact of c-Rel on myosin dynamics. However, muscle and non-muscle cells such as dermal fibroblasts may be regulated differently.

Myofibroblasts resemble smooth muscle cells in their expression and cytoskeletal inclusion of αSMA [32]. However, in contrast to smooth muscle cells the former are permanently contractile presumably also through activation of the Rho/ROCK/myosin light chain phosphatase pathway [40]. Thus, our results described here, together with the outlined molecular details, could be a good starting point for further mechanistic studies on the contractility of myofibroblasts in fibrotic diseases.

Another result, which at first glance seems somewhat curious, was that c-Rel suppression apparently reduced the contractility of fibroblasts in "relaxed", but not in "stressed" gels. On closer examination, however, there appear to be important differences between these two conditions: Former analyses revealed that fibroblasts in “relaxed” matrices have low proliferative activity and a generally pro-inflammatory phenotype, whereas fibroblasts in “stressed” matrices show more myofibroblast traits such as αSMA expression (which was not affected by c-Rel suppression in our experiments) as well as higher contractility [29]. A global influence on the entire fibrotic “machinery” does not seem to occur, because in our models we could not find a general influence of c-Rel suppression on fibrotic markers. Therefore it seems conceivable that c-Rel performs its main function in a “pre-fibrotic” state of fibroblasts, which the "relaxed" matrices may simulate in vitro. If this hypothesis can be confirmed in vivo in future studies, a therapeutic modulation of c-Rel could influence early inflammatory phases of fibrosis and thus have a preventive effect. In any case, we could show that NF-кB c-Rel is an interesting candidate influencing an essential adhesion marker and thus cellular processes in skin fibroblasts with potential consequences for the pathophysiology of fibrosis and/or fibrotic diseases.

## Supplementary Information

Below is the link to the electronic supplementary material.

Supplementary file1 (DOCX 231 KB)

## Data Availability

Not applicable.
