# Supplementary material for: NF-кB c-Rel modulates pre-fibrotic changes in human fibroblasts
Source: Arch Dermatol Res. 2021 Dec 9;314(10):943–51. doi: 10.1007/s00403-021-02310-2 (PMC9522690; doi:10.1007/s00403-021-02310-2)
Supplement: Supplementary file 1 — Supplementary file1 (DOCX 231 KB) [file 403_2021_2310_MOESM1_ESM.docx]

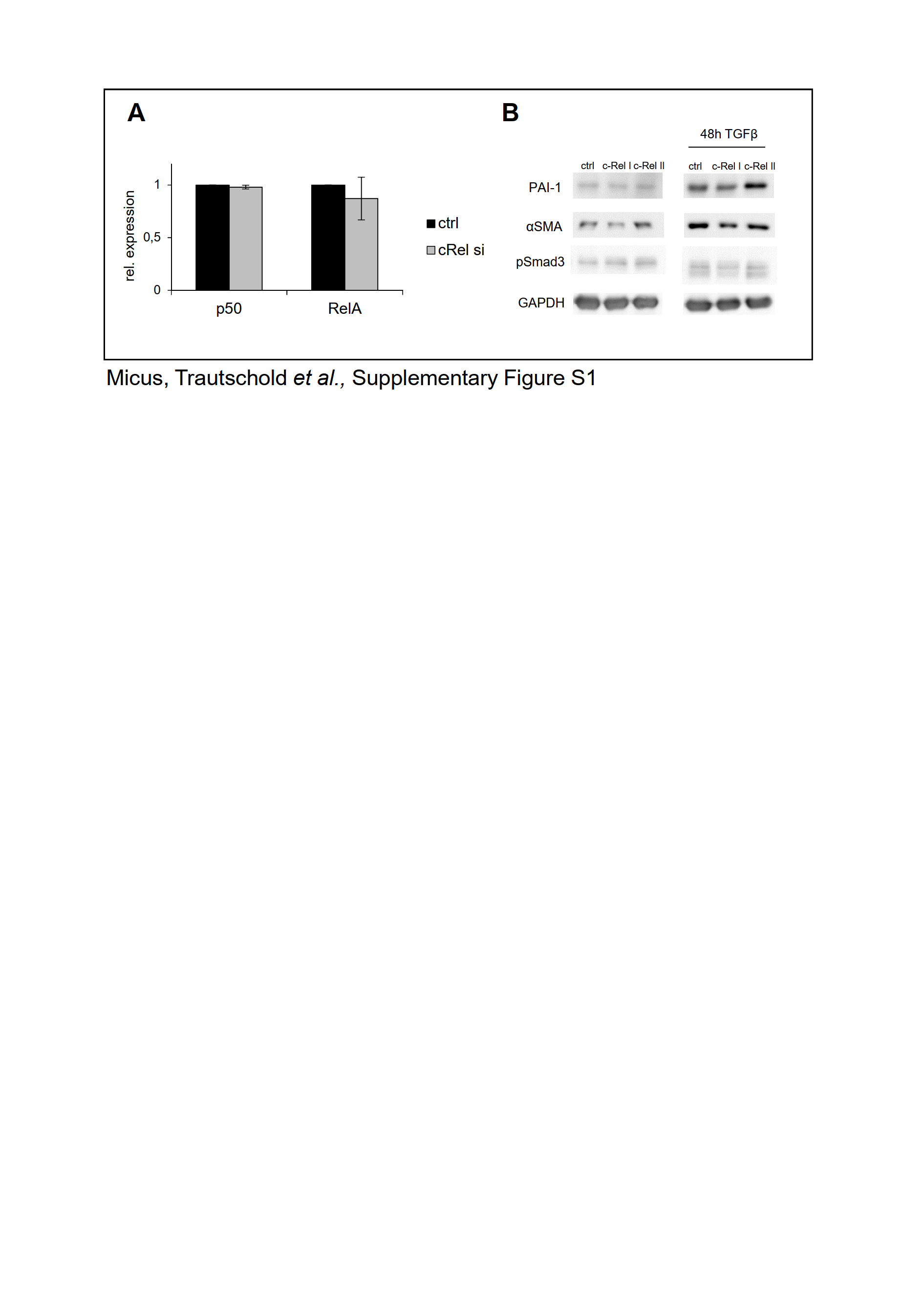


**Supplementary Figure 1: A)** qPCR of p50 and p65 following ctrl and c-Rel II siRNA transfection showing no impact on mRNA expression of either subunit. Relative mean values of three independent experiments are shown, error bars = SEM. **B)** Western blotting of pSmad3, PAI-1 and αSMA following ctrl, c-Rel I and c-Rel II siRNA transfection after 72 h. The experiment shown is representative of three independent experiments showing similar results. GAPDH served as loading control.
